# Propofol induces a metabolic switch to glycolysis and cell death in a mitochondrial electron transport chain-dependent manner

**DOI:** 10.1371/journal.pone.0192796

**Published:** 2018-02-15

**Authors:** Chisato Sumi, Akihisa Okamoto, Hiromasa Tanaka, Kenichiro Nishi, Munenori Kusunoki, Tomohiro Shoji, Takeo Uba, Yoshiyuki Matsuo, Takehiko Adachi, Jun-Ichi Hayashi, Keizo Takenaga, Kiichi Hirota

**Affiliations:** 1 Department of Anesthesiology, Kansai Medical University, Hirakata, Japan; 2 Department of Human Stress Response Science, Institute of Biomedical Science, Kansai Medical University, Hirakata, Japan; 3 Department of Anesthesiology, Tazuke Kofukai Medical Institute Kitano Hospital, Osaka, Japan; 4 University of Tsukuba, Tsukuba, Japan; 5 Department of Life Science, Shimane University Faculty of Medicine, Izumo, Japan; University of PECS Medical School, HUNGARY

## Abstract

The intravenous anesthetic propofol (2,6-diisopropylphenol) has been used for the induction and maintenance of anesthesia and sedation in critical patient care. However, the rare but severe complication propofol infusion syndrome (PRIS) can occur, especially in patients receiving high doses of propofol for prolonged periods. *In vivo* and *in vitro* evidence suggests that the propofol toxicity is related to the impaired mitochondrial function. However, underlying molecular mechanisms remain unknown. Therefore, we investigated effects of propofol on cell metabolism and death using a series of established cell lines of various origins, including neurons, myocytes, and trans-mitochondrial cybrids, with defined mitochondrial DNA deficits. We demonstrated that supraclinical concentrations of propofol in not less than 50 μM disturbed the mitochondrial function and induced a metabolic switch, from oxidative phosphorylation to glycolysis, by targeting mitochondrial complexes I, II and III. This disturbance in mitochondrial electron transport caused the generation of reactive oxygen species, resulting in apoptosis. We also found that a predisposition to mitochondrial dysfunction, caused by a genetic mutation or pharmacological suppression of the electron transport chain by biguanides such as metformin and phenformin, promoted propofol-induced caspase activation and cell death induced by clinical relevant concentrations of propofol in not more than 25 μM. With further experiments with appropriate *in vivo* model, it is possible that the processes to constitute the molecular basis of PRIS are identified.

## Introduction

Since its introduction into clinical practice in 1986, propofol (2,6-diisopropylphenol) has been used for the induction and maintenance of anesthesia and sedation in critical patient care [1]. Although propofol is considered a safe agent for anesthesia and sedation, propofol infusion syndrome (PRIS), a rare but severe complication can occur, especially in patients receiving high doses of the anesthetic for prolonged periods [2, 3]. However, the exact etiology of PRIS remains unclear. It is characterized by the development of metabolic acidosis (lactic acidosis), rhabdomyolysis, hyperkalemia, hepatomegaly, renal failure, arrhythmia, and progressive cardiac failure [2]. There is a strong association between PRIS and propofol infusion at doses greater than 4 mg/kg/h and an exposure duration longer than 48 h. *In vivo* and *in vitro* evidence suggests that PRIS is related to impaired mitochondrial function [4–6]. Preexisting mitochondrial disorders may increase the risk of developing PRIS [4, 7, 8]. Moreover, studies using isolated mitochondria have demonstrated that propofol affects mitochondrial respiration. A decrease in the mitochondrial membrane potential (ΔΨm) has been reported in liver mitochondria isolated from rats treated with propofol [9]. An increase of the oxygen consumption rate (OCR) has suggested that propofol acts as an uncoupler in oxidative phosphorylation (OXPHOS) [10]. The incubation of mitochondria isolated from rats treated with high concentrations of propofol (100–400 μM) results in a strong inhibition of the activity of complex I and to a lesser degree, of complexes II and III [6]. Based on the reduction of complex IV activity in skeletal muscles, propofol after metabolism is hypothesized to disrupt mitochondrial respiration [11].

However, precise molecular mechanisms, including the relationship between mitochondrial defects and metabolic reprogramming in the pathophysiology of PRIS, are largely unknown as yet. To investigate underlying cellular and molecular mechanisms of PRIS, we investigated the effects of propofol on cell life and death, oxygen metabolism, and mitochondrial function using cells of various origins, including transmitochondrial cybrids harboring a mitochondrial DNA defect and mutations. We demonstrated that propofol, used within a clinically relevant exposure time, suppressed the mitochondrial function, causes reactive oxygen species (ROS) generation, and induced the metabolic switch from OXPHOS to glycolysis by targeting complexes I, II and III of mitochondria [12]. The data also indicated that predisposition to mitochondrial dysfunction, caused by a genetic mutation or the pharmacological suppression of the electron transport chain (ETC) in mitochondria by biguanides such as metformin and phenformin, promotes propofol-induced caspase activation and cell death.

## Materials and methods

### Reagents

Propofol (2,6-diisopropylphenol), 2,4-diisopropylphenol, dimethyl sulfoxide (DMSO), rotenone, oligomycin, and antimycin A were obtained from Sigma–Aldrich (St. Louis, MO, USA). N,N,N′,N′-tetramethyl-*p*-phenylenediamine (TMPD) was from Wako (Tokyo, Japan). Succinate and ascorbate were obtained from Nakalai Tesque (Kyoto, Japan). Details of the reagents used in this study are provided in S1 Table.

### Cell lines and cell culture

Established cell lines derived from human neuroblastoma SH-SY5Y cells (ATCC® CRL-2266™), cervical carcinoma HeLa cells (ATCC® CCL-2™) and myoblast C2C12 cells (ATCC® CRL-1772) were obtained from American Type Culture Collection (ATCC), Manassas, VA, USA. The cells were maintained in RPMI 1640 medium supplemented with 10% fetal bovine serum, 100 U/mL penicillin, and 0.1 mg/mL streptomycin. The mouse cell lines and trans-mitochondrial cybrids cells are listed in S2 Table and S3 Table. P29 cells originated from Lewis lung carcinoma (C57BL/6 mouse strain), and B82 cells are fibrosarcoma cells derived from the L929 fibroblast cell line (C3H/An mouse strain) [13–15]. Parental P29 cells, ρ0 cells, and the transmitochondrial cybrids were grown in Dulbecco’s modified Eagle’s medium (DMEM) supplemented with pyruvate (0.1 mg/mL), uridine (50 mg/mL), and 10% fetal bovine serum. We isolated ρ0 cells by treating parental P29 cells with 1.5 mg/mL ditercalinium, an antitumor *bis*-intercalating agent. Enucleated cells of mtDNA donors were prepared by pretreatment with cytochalasin B (10 μg/mL) for 2 min, followed by centrifugation at 7,500 ×*g* for 10 min [14]. The resultant cytoplasts were fused with ρ0 cells using polyethylene glycol. The trans-mitochondrial cybrids (S3 Table) were isolated in a selection medium that allows exclusive growth of the cybrids [14]. All the P29 cells and the corresponding derivatives are generated and maintained by Dr. Keizo Takenaga (Shimane University Faculty of Medicine, Izumo, Japan) and Dr. Jun-Ichi Hayashi (Tsukuba University, Tsukuba, Japan).

### Propofol treatment

Propofol was diluted with dimethyl sulfoxide and used. The same volume of DMSO was applied to each well or dish as a vehicle control in 0.5% v/v with a maximum. The concentrations and durations of treatment were described in each figure and each legend.

### Analysis of cell death

A previously established protocol was used [16, 17]. Briefly, apoptosis was measured using an annexin V–FITC apoptosis detection kit (BioVision, Milpitas, CA, USA), according to the manufacturer’s instructions. For the analysis, cells were seeded into 6-well plates (3 × 10^5^ cells/well) and incubated overnight. On the following day, the cells were treated with the various concentrations of the appropriate drug for varying times and harvested by centrifugation at 250 x*g* for 3 min. The cell pellets were resuspended in a mixture comprised of 500 μL of binding buffer, 5 μL of annexin V–FITC, and 5 μL of propidium iodide (PI 50 μg/mL). The suspensions were incubated for 5 min at room temperature in the dark and analyzed using a FACSCalibur™ flow cytometer (BD Biosciences, San Jose, CA, USA) equipped with the CellQuest Pro™ software version 5.2 (BD Biosciences, San Jose, CA, USA). The data were evaluated using the FlowJo™ version 9.9.4 software (TreeStar, Ashland, OR, USA)) as shown in S4 Fig.

### Caspase-3/7 and caspase-9 activity assays

Activities of caspase-3/7 and caspase-9 were assessed using an Apo-ONE™ homogeneous caspase-3/7 assay kit (Promega, Madison, WI, USA) and a Caspase-Glo™ 9 assay kit (Promega), respectively, according to the manufacturer’s protocols [16, 17]. Briefly, cells were seeded into 96-well plates (2 × 10^4^ cells/well) and incubated overnight. On the following day, the cells were treated with the indicated concentrations of the appropriate drug for varying times. After the treatment, 100 μL of the Apo-ONE™ caspase-3/7 reagent was added to each well. The plates were incubated at room temperature for 1 h, and the luminescence of each well was measured using an EnSpire™ multimode plate reader (PerkinElmer, Waltham, MA, USA). Caspase activity was calculated by comparing the levels of luminescence of the treated cells with that of the control cell population incubated without drugs, which was defined as 100%.

### Determination of mitochondrial membrane potential

The mitochondrial membrane potential (ΔΨm) was determined by flow cytometry using a MitoPT™ JC-1 assay kit (ImmunoChemistry Technologies, Bloomington, MN, USA), according to the manufacturer’s instructions [16]. For the analysis, cells were seeded into 6-well plates (3 × 10^5^ cells/well) and cultured overnight. On the following day, the cells were treated with the indicated concentrations of the appropriate drug for 6 h and then pelleted by centrifugation at 250 x*g* for 3 min. The cells were then resuspended in JC-1, incubated at 37°C for 15 min in the dark, and collected by centrifugation at 1,200 x*g* for 3 min. The cell pellets were resuspended in 500 μL of assay buffer. The samples were subsequently analyzed using a FACSCalibur™ flow cytometer (BD Biosciences) equipped with the CellQuest Pro™ software version 5.2 (BD Biosciences) for the detection of red JC-1 aggregates (590 nm emission) or green JC-1 monomers (527 nm emission). The data were evaluated using the FlowJo version 7.6.3 software (TreeStar), then exported to Microsoft Office Excel, and subsequently analyzed using the statistical application GraphPad™ Prism version 7.0b (GraphPad Software, Inc. La Jolla, CA USA).

### LDH-based cytotoxicity assay

Cytotoxicity was measured using a CytoTox-ONE™ kit (Promega) as described previously [16, 17]. Briefly, cells were cultured overnight in 96-well plates (2 × 10^4^ cells/well) and treated with the indicated drug for varying times. After adding 20 μL of the CytoTox-ONE™ reagent was added to each well, and the plates were incubated at 22°C for 10 min. The reaction was terminated by adding 50 μL of the stop solution, and the fluorescence was recorded at an excitation wavelength of 560 nm and an emission wavelength of 590 nm using an EnSpire™ multimode plate reader (PerkinElmer). The percentage of cell death was determined by comparing the release of LDH (based on fluorescence measurement) in each treatment group with that of the positive control treated with the lysis solution, which was defined as 100%. The level of LDH release from untreated cells (negative control) was defined as 0%.

### Cell growth MTS assay

Cell growth was assessed using a CellTiter 96 AQueous One Solution Cell Proliferation Assay™ with MTS (Promega) [16, 17]. Briefly, cells were seeded into 96-well plates (2 × 10^4^ cells/well) and cultured overnight. On the following day, the cells were treated with the indicated concentrations of the appropriate drugs for varying times. After the treatment, 20 μL of the CellTiter 96 AQueous One Solution™ reagent was added to each well, the plates were incubated at 37°C for 1 h, and the absorbance of each sample was measured using an iMark™ microplate reader (Bio-Rad, Hercules, CA, USA) at a wavelength of 490 nm. Cell viability was calculated by comparing the absorbance of treated cells with that of the control cells incubated without drugs, which was defined as 100%.

### Measurement of ROS generation

ROS generation was detected with 2′,7′-dichlorodihydrofluorescine diacetate (DCFH-DA) (Molecular Probes, Eugene, OR, USA). Briefly, cells cultured in 35-mm-diameter glass-bottom culture dishes (MatTek, Ashland, MA, USA) were incubated with 10 μM DCFH-DA for 10 min at 37°C in serum-free DMEM, then washed twice with Dulbecco’s phosphate-buffered saline, and analyzed using FACSCalibur flow cytometer (BD Biosciences). The mean fluorescence intensity was analyzed using the CellQuest software version 5.2 (BD Biosciences).

### Cellular oxygen consumption and extracellular acidification measurement

Cellular OCR and ECAR were determined with the XF Cell Mito Stress Test™ and XF Glycolysis Stress Test™, respectively, using an XFp Extracellular Flux Analyzer™ (Seahorse Bioscience, USA) [17]. Cells (2 × 10^4^ cells/well) were seeded into an XFp cell culture microplate, and OCR was assessed in glucose-containing XF base medium according to the manufacturer’s instructions. The sensor cartridge for the XFp analyzer was hydrated in a 37°C non-CO_2_ incubator on the day before the experiment. Each extracellular flux assay included two control wells of medium alone (no cells) to provide the blanks for background correction. They are used to normalize the data to the background signal derived from non-cell-mediated process. For the OCR assay, injection port A on the sensor cartridge was loaded with oligomycin (complex V inhibitor, final concentration 1 μM), port B was loaded with carbonyl cyanide-4-(trifluoromethoxy) phenylhydrazone (FCCP, final concentration 2 μM), and port C was loaded with rotenone/antimycin A (inhibitors of complex I and complex III, final concentration 0.5 μM each). During sensor calibration, cells were incubated in a 37°C non-CO_2_ incubator in 180 μL of assay medium (XF base medium with 25 mM glucose, 1 mM pyruvate, and 2 mM L-glutamine, pH 7.4). The plate was immediately placed into the calibrated XFp extracellular flux analyzer for the Mito Stress test. The assay parameters were calculated as follows: OCR (basal respiration) = (last rate measurement before oligomycin injection) − (minimum rate measurement after rotenone/antimycin-A injection); OCR (maximal respiration) = (maximum rate measurement after FCCP injection) − (minimum rate measurement after rotenone/antimycin A injection); OCR (non-mitochondrial respiration) = (minimum rate measurement after rotenone/antimycin A injection); proton leak = (minimum rate measurement after oligomycin injection)–(non-mitochondrial respiration). For the ECAR assay, injection port A on the sensor cartridge was loaded with 10 mM glucose. During the sensor calibration, cells were incubated in a 37°C non-CO_2_ incubator in 180 μL of assay medium (XF base medium with 2 mM L-glutamine, pH 7.4). The plate was immediately placed into the calibrated XFp extracellular flux analyzer™ for the Glycolysis Stress test. Oligomycin (1 μM) and 50 mM 2-deoxy-D-glucose were loaded for the measurement. ECAR was calculated as follows: ECAR (glycolysis) = (maximum rate measurement after glucose injection) − (last rate measurement before glucose injection).

### Measurement of oxygen consumption in permeabilized cells

The activity of individual respiratory chain complexes was evaluated in permeabilized cells [18, 19]. Briefly, cells were washed with mitochondrial assay solution (MAS) buffer (220 mM mannitol, 70 mM sucrose, 10 mM KH_2_PO_4_, 5 mM MgCl_2_, 2 mM HEPES, 1 mM EGTA, 0.2% fatty acid-free bovine albumin, adjusted to pH 7.2 with KOH), and the medium was replaced with MAS buffer supplemented with 10 mM pyruvate, 1 mM malate, 4 mM ADP, and 1 nM plasma membrane permeabilizer™. The cells were then loaded into the XFp analyzer to measure respiration rates using cycles of 30 s mixing/30 s waiting/2 min measurement. Protocol A: After the measurement of pyruvate-driven respiration, rotenone (final concentration 2 μM) was injected through port A to halt the complex I-mediated respiratory activity. Next, succinate (10 mM) was injected through port B to donate electrons at complex II, bypassing complex I inhibition. The addition of antimycin A (2 μM) via port C inhibited complex III, and N,N,N′,N′-tetramethyl-*p*-phenylenediamine (TMPD 0.1 mM), combined with ascorbate (10 mM), was subsequently injected through port D to measure complex IV activity. Protocol B: As an alternative approach, cells were initially supplemented with pyruvate to measure complex I activity. After injection of rotenone, duroquinol was injected to stimulate complex III-mediated respiration.

### Statistical analysis

All experiments were repeated at least twice, and each sample was evaluated in triplicate. Representative data, expressed as the mean ± SD, are shown. Differences between treatment groups were evaluated by one-way or two-way ANOVA, followed by appropriate multiple comparison test using GraphPad Prism version 7.0b (GraphPad Software). *P*-values of < 0.05 were considered statistically significant (S1 Data).

## Results

### Propofol induced cell death and activation of caspases in a concentration- and time-dependent manner

**Fig 1:**
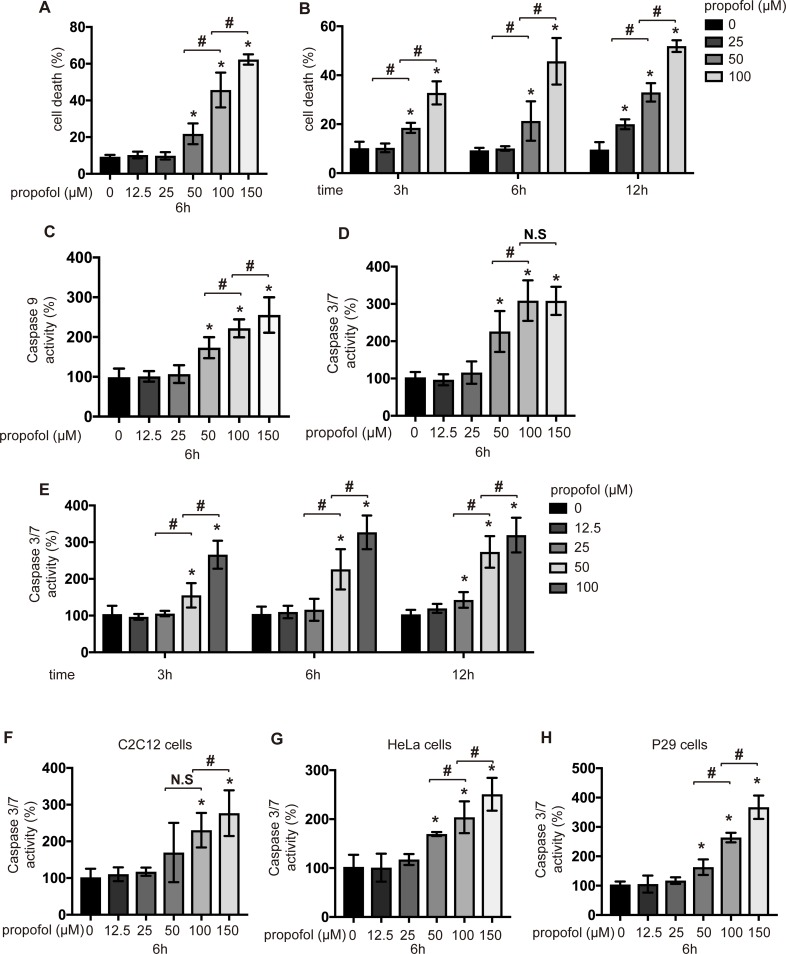
Propofol induced cell death and caspase activation in a concentration- and time-dependent manner. SH-SY5Y cells were exposed to the indicated concentrations (12.5, 25, 50, 100, or 150 μM) of propofol for 6 h (A) and 3, 6, and 12 h (B). Cells were harvested, and percentages of cell death were measured by flow cytometry. The ratio of PI-positive and/or annexin V-positive cells [(Q1 + Q2 + Q4)/(Q1 + Q2 + Q3 + Q4)] was used to calculate the percentage of dead cells (A and B) (S1 Fig) (n = 3). SH-SY5Y cells were exposed to the indicated concentrations (12.5, 25, 50, 100, or 150 μM) of propofol for 6 h (C and D) and 3, 6, and 12 h (E). Caspase-9 (n = 5) (C) and caspase-3/7 (n = 5) (D and E) activities were assayed in each treatment group at different time points. (F, G and H) C2C12 cells (F), HeLa cells (G) and P29 cells (H) were exposed to the indicated concentrations (12.5, 25, 50, 100 or 150 μM) of propofol for 6 h. The graphical depiction of caspase-3/7 activity is shown (n = 3). Data presented are expressed as the mean ± SD. Differences between treatment groups were evaluated by one-way ANOVA, followed by Tukey's multiple comparison test (A, C, D, F, G and H), or by two-way ANOVA, followed by Tukey's multiple comparison test (B and E). *p < 0.05 compared to the control cell population (incubation for 0 h, no treatment).

**Fig 2:**
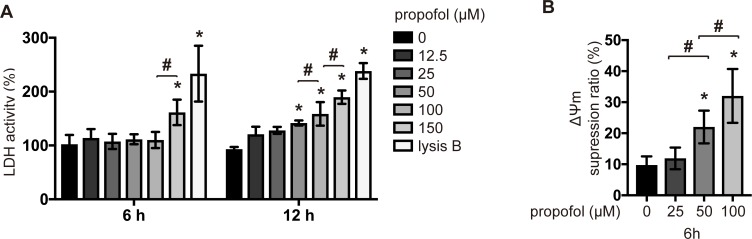
Propofol induced cell death and decreased mitochondrial membrane potential in a concentration- and time-dependent manner. (A) SH-SY5Y cells were exposed to the indicated concentrations (12.5, 25, 50, 100, or 150 μM) of propofol for 6 h and 12 h. LDH release was assayed in culture supernatants (n = 3). Treatment with lysis buffer served as a control. (B) Average mitochondrial membrane potential (ΔΨm) of untreated SH-SY5Y cells and SH-SY5Y cells treated with the indicated concentrations (25, 50, or 100 μM) of propofol (n = 3) for 6 h. Values indicate the ratio [Q2/(Q2 + Q4)] of green JC-1 monomers (527 nm emission) to red aggregates (590 nm emission). Data are expressed as the mean ± SD. Differences between treatment groups were evaluated by two-way ANOVA, followed by Tukey's multiple comparison test (A), or by one-way ANOVA, followed by Tukey's multiple comparison test (B). *p < 0.05 compared to the control cell population (incubation for 0 h, no treatment).

To determine whether propofol induces the cell death, we examined the effects of propofol concentration and incubation time on cell death by flow cytometry. Concentrations of propofol equal to or greater than 50 μM induced the cell death within 6 h (Fig 1A and S1 Fig). Interestingly, 25 μM propofol induced the cell death only after 12 h of incubation (Fig 1B). Next, activities of caspase-9 and caspase-3/7 were evaluated. Propofol at concentrations equal to or greater than 50 μM activated caspase-9 (Fig 1C). Caspase-9 activation initiates the caspase cascade through caspase-3 and caspase-7 activation [20]. Caspase-3/7 was activated by treatment with propofol at a concentration of 50 μM or greater within 6 h (Fig 1D). Importantly, 25 μM propofol, which did not induce caspase-3/7 activation within 6 h (Fig 1E), induced the activation at 12 h (Fig 1E). Propofol at a concentration of 150 μM led to the detachment of cells from the wells during the 12-h incubation. Finally, the effects of propofol were investigated in cells of different origins, including mouse myoblast C2C12 cells (Fig 1F), human cervical cancer HeLa cells (Fig 1G), and Lewis lung carcinoma P29 cells (Fig 1H). Similar to its effects on SH-SY5Y cells, 50 and 100 μM propofol but not 12.5 or 25 μM propofol induced caspase-3/7 activation in the other cell lines within 6 h. Next, LDH was investigated (Fig 2A). Within 6 h, only propofol at a concentration of 150 μM increased the LDH release. However, after 12 h of incubation, LDH release increased significantly not only at 150 μM propofol, but also at 50 and 100 μM propofol. The measurement of the mitochondrial membrane potential showed that propofol at concentrations equal to or greater than 50 μM decreased the membrane potential within 6 h (Fig 2B). In addition, at concentrations greater than 50 μM, propofol suppressed the cell viability, measured by an MTS [3-(4,5-dimethylthiazol-2-yl)-5-(3-carboxymethoxyphenyl)-2-(4-sulfophenyl)-2*H*-tetrazolium] assay, within 6 h (Fig A in S2 Fig). 2,4-diisopropylphenol is an isomeric form of propofol, which does not show a hypnotic effect. We tested the effects of 2,4-diisopropylphenol on caspase-3/7 activation in SH-SY5Y cells. Similar to propofol, 2,4-diisopropylphenol induced caspase-3/7 activation within 6 h (Fig B in S2 Fig). Interestingly, 25 μM 2,4-diisopropylphenol could activate caspase-3/7 within 6 h, suggesting that 2,4-diisopropylphenol is more toxic than propofol.

### Propofol suppressed oxygen metabolism and induced ROS generation

**Fig 3:**
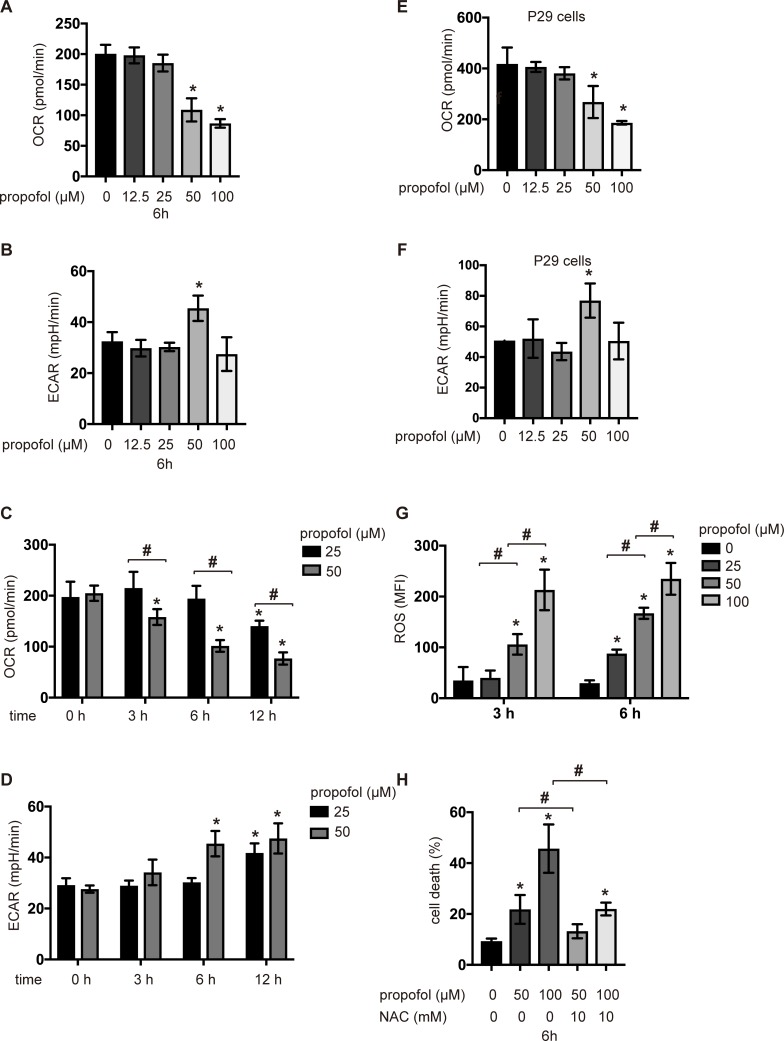
Oxygen metabolism and ROS generation in SH-SY5Y cells and P21 cells treated with propofol. OCR (A, C, and E) and ECAR (B, D, and F) in SH-SY5Y cells or P29 cells exposed to the indicated concentrations of propofol (12.5, 25, 50, or 100 μM) for 6 h (A, B, E and F, respectively) or 0, 3, 6, or 12 h (C and D). Data presented are expressed as the mean ± SD. Differences between treatment groups were evaluated by one-way ANOVA, followed by Dunnett’s multiple comparison test (A, B, E and F), or by two-way ANOVA, followed by Dunnett’s multiple comparison test (C and D). (G) ROS production was measured in SH-SY5Y cells exposed to 25, 50, or 100 μM propofol (n = 3) for 3 h or 6 h. (H) SH-SY5Y cells were exposed to the indicated concentrations (50 or 100 μM) of propofol for 6 h with or without treatment with 10 mM *N*-acetylcysteine. Cells were harvested, and percentages of cell death were measured by flow cytometry. MFI: median fluorescence intensity; NAC: *N*-acetylcysteine. Data presented are expressed as the mean ± SD. Differences between treatment groups were evaluated by two-way ANOVA, followed by Tukey's multiple comparison test (G), or by one-way ANOVA, followed by Tukey's multiple comparison test (H). *p < 0.05 compared to the control cell population.

We investigated the effects of propofol on oxygen metabolism and glycolysis in SH-SY5Y cells by assaying OCR and the extracellular acidification rate (ECAR), which is a surrogate index for glycolysis. SH-SY5Y cells were preincubated with the indicated concentrations of propofol for the indicated periods. OCR was significantly suppressed by the treatment with 50 and 100 μM propofol for 6 h (Fig 3A and 3C and Fig A, C and E-H in S3 Fig). Accordingly, the ECAR levels significantly increased upon the treatment with 50 μM propofol (Fig 3B and 3D and Fig B and D in S3). Propofol at a concentration of 25 μM, which exerted no significant effects within 6 h, suppressed OCR (Fig 3C) and enhanced ECAR (Fig 3D) after 12 h of incubation. In addition to SH-SY5Y cells, 50 and 100μM propofol suppressed OCR (Fig 3E) and increased ECAR (Fig 3F) in P29 cells after 6 h. These results indicate that propofol affects oxygen metabolism in mitochondria. It has been reported that disturbance in mitochondrial ETC leads to the generation of ROS in cells [21, 22]. ROS generation was observed in SH-SY5Y cells in response to propofol exposure within 3 and 6 h (Fig 3G). Cell death induced by 50 or 100 μM propofol was suppressed by treatment with 10 mM of the antioxidant *N*-acetylcysteine (Fig 3H).

### Involvement of mitochondria in propofol-induced cell death and caspase activation

**Fig 4:**
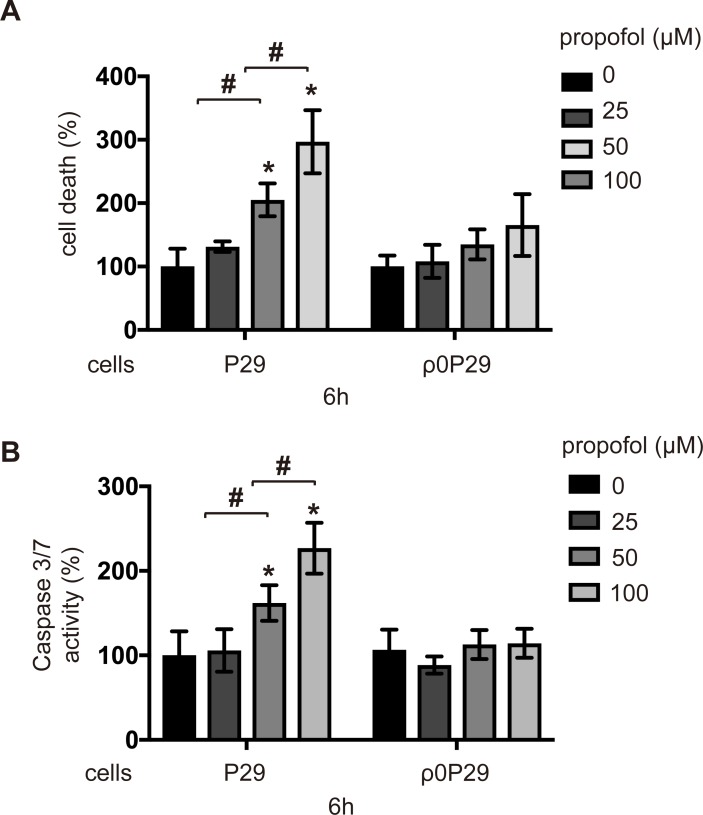
Involvement of functional mitochondria in propofol-induced caspase activation and cell death. P29 and ρ0P29 cells lacking mtDNA were exposed to the indicated concentrations (25, 50 or 100 μM) of propofol for 6 h. (A) Cells were harvested, and percentages of cell death were measured by flow cytometry. The ratio of PI-positive and/or annexin V-positive cells [(Q1 + Q2 + Q4)/(Q1 + Q2 + Q3 + Q4)] was used to calculate the percentage of dead cells (S1 Fig) (n = 3). (B) Caspase-3/7 activity in each treatment group (n = 3) at 6 h. Differences between treatment groups were evaluated by one-way ANOVA, followed by Tukey's multiple comparison test. *p < 0.05 compared to the control cell population; #*p* < 0.05 compared to the indicated experimental groups.

As shown in Fig 3, propofol affected mitochondrial ETC and intracellular oxygen metabolism in SH-SY5Y cells and P29 cells. To examine the involvement of mitochondria in propofol-induced cell death, P29 and ρ0P29 cells were exposed to the indicated concentrations of propofol for 6 h, and then cell death (Fig 4A) and caspase-3/7 activity (Fig 4B) were assayed. Both cell death and caspase-3/7 assays indicated that, unlike P29 cells, ρ0P29 cells were completely resistant to 50 μM and partially resistant to 100 μM propofol after 6 h of incubation (Fig 4A and 4B). Together with results showing that propofol affects mitochondrial ETC, these results strongly suggests that mitochondria play a critical role and are one of the targets in propofol-induced cell death.

### Effects of propofol on ETC complex-dependent OCR

**Fig 5:**
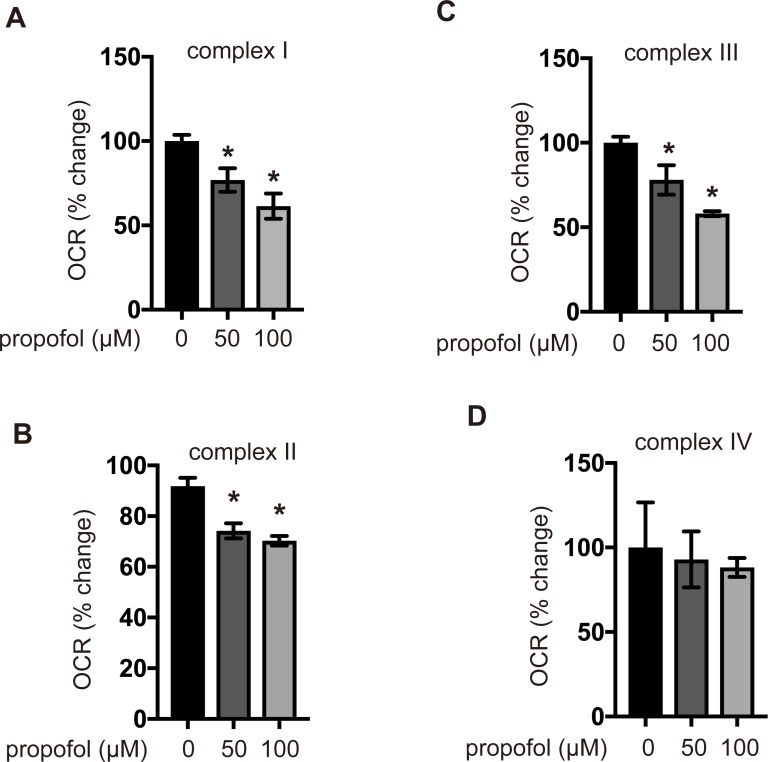
Effects of propofol on OCR driven by each complex of the mitochondrial ETC. Representative OCR traces of mitochondrial respiration using protocol A (Fig A and B in S4 Fig) and protocol B (Fig C and D in S4 Fig). Mitochondrial ETC-mediated OCR, driven by complexes I (A), II (B), III (C), and IV (D), were assayed using an extracellular flux analyzer. SH-SY5Y cells were exposed to 50 or 100 μM propofol for 6 h and subjected to the assay. Differences between treatment groups were evaluated by one-way ANOVA, followed by Dunnett’s multiple comparison test. *p < 0.05 compared to the control cell population.

Further, we examined OCR, which depends on the activity of mitochondrial respiratory chain complexes I–IV in membrane-permeabilized and intact cells, using an extracellular flux analyzer. Traces of OCR due to mitochondrial respiration were detected using protocol A (Fig 5A, 5B and 5D and Fig A and B in S4 Fig) and protocol B (Fig 5C and Fig C and D in S4 Fig). The results indicated that propofol significantly suppressed the complex I-, the complex II- and complex III-dependent OCR but not complex IV-dependent OCR.

### Mitochondrial ETC inhibitors synergistically enhanced propofol toxicity

**Fig 6:**
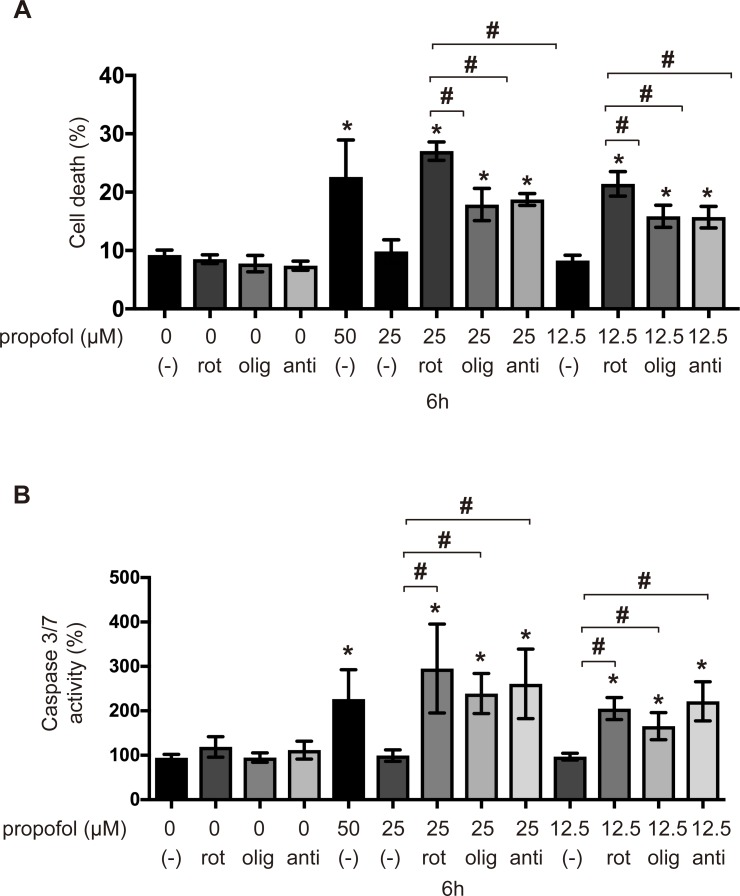
Synergistic effects of propofol and mitochondrial ETC inhibitors on caspase activity and cell death. Levels of caspase-3/7 activity and cell death in SH-SY5Y cells treated with propofol and mitochondrial ETC inhibitors. Cells were treated with 12.5 or 25 μM propofol and with either 100 nM rotenone, 4 μM oligomycin, or 25 μg/mL antimycin A for 6 h and subjected to (A) a cell death assay and (B) a caspase-3/7 activity assay (n = 3). Percentages of cell death were measured by flow cytometry. The ratio of PI-positive and/or annexin V-positive cells [(Q1 + Q2 + Q4)/(Q1 + Q2 + Q3 + Q4)] was used to calculate the percentage of dead cells (S1 Fig) (n = 3). All data are expressed as the mean ± SD. Differences between treatment groups were evaluated by one-way ANOVA, followed by Tukey's multiple comparison test. *p < 0.05 compared with control cells (no treatment); #*p* < 0.05 compared with the indicated groups. rot: rotenone; olig: oligomycin; anti: antimycin A.

We investigated the effects of several ETC inhibitors on the propofol-induced cell death in SH-SY5Y cells. Cell death was not induced by 100 nM rotenone, 4 μM oligomycin, 25 μg/mL antimycin A or propofol alone at 12.5 or 25 μM within 6 h (Fig 6A). However, 12.5 and 25 μM propofol induced cell death in the presence of rotenone, antimycin A, and oligomycin (Fig 6A). Similarly, 12.5 and 25 μM propofol with rotenone, oligomycin, and antimycin A induced caspase-3/7 activity within 6 h (Fig 6B). These results indicate that the synergistic inhibition of mitochondria by a combination of propofol and ETC inhibitors induces cell death, even at clinically relevant concentrations of propofol within 6 h.

### Genetic predisposition to mitochondrial dysfunction increased propofol-induced caspase activation and cell death

**Fig 7:**
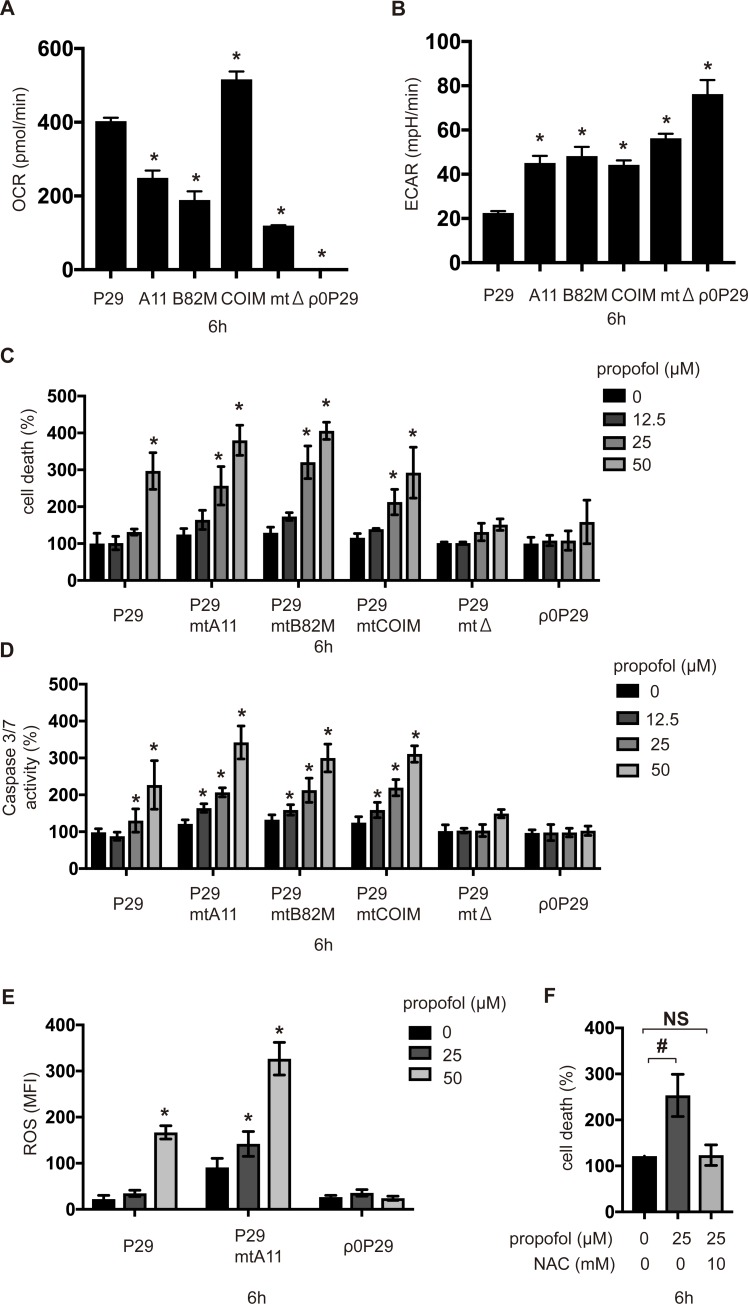
Effects of propofol on caspase activity and cell death in various transmitochondrial cybrid cells. (A) OCR and (B) ECAR of P29, its cybrid cells, and ρ0P29 cells. (C and D) P29, its cybrid cells, and ρ0P29 cells were exposed to the indicated concentrations (12.5, 25, or 50 μM) of propofol for 6 h. Cells were harvested, and percentages of cell death were measured by flow cytometry. The ratio of PI-positive and/or annexin V-positive cells [(Q1 + Q2 + Q4)/(Q1 + Q2 + Q3 + Q4)] was used to calculate the percentage of dead cells (n = 3) (S1 Fig) (C). Caspase-3/7 activity in each treatment group were assayed (n = 3) (D). (E) P29, P29mtA11 and ρ0P29 cells were exposed to 25 or 50 μM propofol for 6 h and subjected to ROS assay. (F) P29mtA11 cells were exposed to 25 μM propofol with or without 10 mM NAC for 6 h. Cells were harvested, and percentages of cell death were measured by flow cytometry. Data presented in (A–F) are expressed as the mean ± SD. Differences between treatment groups were evaluated by one-way ANOVA, followed by Tukey's multiple comparison test (A and B), by Dunnett’s multiple comparison (E and F) or by two-way ANOVA, followed by Tukey's multiple comparison test (C and D). *p < 0.05 compared to the control cell population; #*p* < 0.05 compared with the indicated groups. A11: P29mtA11 cells; B82M: P29mtB82M cells; COIM: P29mtCOIM cells; mtΔ: P29mtΔ cells.

Further, oxygen metabolism profiles of the transmitochondrial cybrid clones were examined (Fig 7A and 7B). These transmitochondrial cybrids were exposed to 12.5, 25 or 50 μM propofol for 6 h to investigate the cell death and caspase-3/7 activation. Interestingly, in contrast to parental P29 cells, flow cytometry analysis indicated that 25 μM propofol induced the cell death within 6 h in P29mtA11, P29mtB82M, and P29mtCOIM cells. 12.5 μM propofol did not induce the cell death within 6 h (Fig 7C). Caspase-3/7 activation was also induced in these cells even with 12.5 μM propofol (Fig 7D). However, P29mtΔ cells, similar to ρ0P29 cells, were completely resistant to 50 μM propofol (Fig 7C and 7D). Thus, our experimental results indicated that cells harboring genetic mutations in mtDNA (*ND6* in complex I or *COI* in complex IV) were more susceptible to propofol-induced cell death.

ROS generation was investigated in P29 cells, P29mtA11 cells, and ρ0P29 cells in response to propofol exposure (Fig 7E). Cells were exposed to 25 or 50 μM propofol for 6 h (Fig 7E). An increased ROS level was found in P29mtA11 cells compared to that in P29 cells. Propofol exposure at 25 μM, which did not induce ROS generation in P29 cells, had a potent effect on P29mtA11 cells. However, no ROS generation was observed in ρ0P29 cells, even at propofol concentrations lesser than 50 μM. Accordingly, cell death induced by 25 μM propofol was suppressed by treatment with 10 mM NAC in P29mtA11 cells (Fig 7F).

### Pharmacological suppression of mitochondrial ETC increased propofol-induced cell death and caspase activation

**Fig 8:**
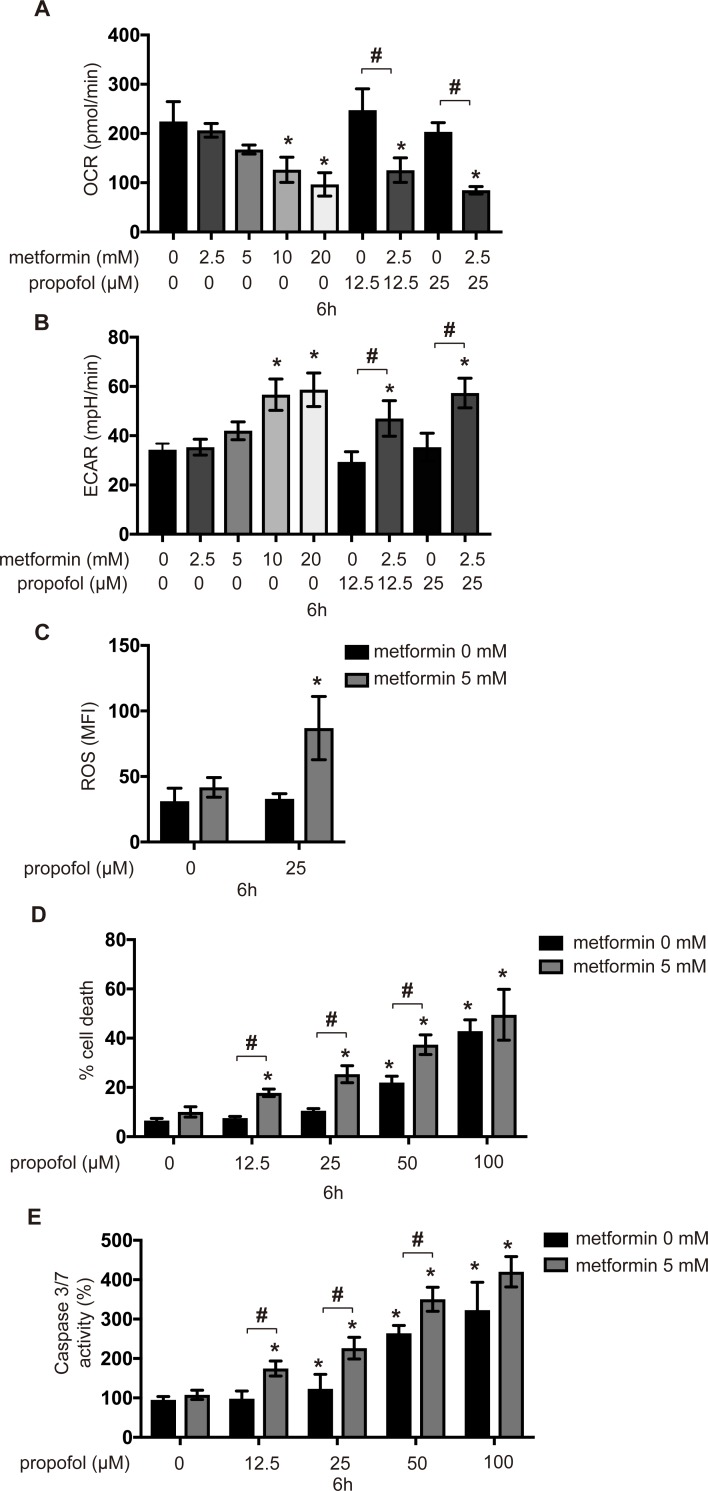
Synergistic effects of propofol and metformin on caspase activity and cell death. (A) OCR and (B) ECAR of SH-SY5Y cells exposed to the indicated concentrations of metformin (2.5, 5, 10, or 20 mM) with or without propofol for 6 h. (C) SH-SY5Y cells were exposed to 25 μM propofol with or without 5 mM metformin for 6 h, and ROS production was determined (n = 3). (D and E) SH-SY5Y cells were exposed to the indicated concentrations (12.5, 25, 50, or 100 μM) of propofol with or without 5 mM metformin for 6 h. (D) Cells were harvested, and percentages of cell death were measured by flow cytometry. The ratio of PI-positive and/or annexin V-positive cells [(Q1 + Q2 + Q4)/(Q1 + Q2 + Q3 + Q4)] was used to calculate the percentage of dead cells (n = 3) (S1 Fig). (E) Caspase-3/7 activity in each treatment group (n = 3). Data presented in (A–E) are expressed as the mean ± SD. Differences between treatment groups were evaluated by one-way ANOVA, followed by Dunnett’s multiple comparison test (A, B, and C), or by two-way ANOVA, followed by Tukey's multiple comparison test (D and E). *p < 0.05 compared to the control cell population; #*p* < 0.05 compared to the indicated experimental groups.

Biguanides, such as metformin and phenformin, are widely used as antihyperglycemics [23]. Metformin and phenformin have also been shown to suppress complex I of ETC, which is used by cells to generate energy [24–28]. To confirm the effect of the blockade of ETC, SH-SY5Y cells were pretreated with 2.5–20 mM metformin or 5–15 μM phenformin for 6 h, with or without 12.5 or 25 μM propofol, and then the cells were tested using the OCR and ECAR assays. Incubation for 6 h with either 2.5 mM metformin or 5 μM phenformin did not affect OCR (Fig 8A and Fig A in S5 Fig) and ECAR (Fig 8B and Fig B in S5 Fig) in SH-SY5Y cells. However, propofol at both 12.5 and 25 μM concentrations significantly decreased OCR (Fig 8A) and increased ECAR (Fig 8B) in the presence of 2.5 mM metformin. Next, we investigated ROS generation after exposure to 25 μM propofol with or without 5 mM metformin. Exposure to 25 μM propofol did not induce ROS generation without metformin. However, when combined with metformin, 25 μM propofol induced ROS generation (Fig 8C). Then, SH-SY5Y cells were tested for cell death (Fig 8D) and caspase 3/7 activation (Fig 8E) after treatment with 5 mM metformin or 5 μM phenformin (Fig C and D in S5 Fig) for 6 h, with or without propofol. Metformin at 5 mM and phenformin at 5 μM increased propofol-induced cell death and caspase-3/7 activation.

## Discussion

In this study, we demonstrated for the first time that propofol, at clinically relevant concentrations and incubation times, alters cellular oxygen metabolism by targeting mitochondrial complexes I, II, and III and induces a cellular metabolic switch from OXPHOS to glycolysis; it also induced ROS generation. Mitochondrial suppression induced death in cell lines of various origins, including trans-mitochondrial cybrids carrying mtDNA with defined pathogenic mutations. Mitochondria play key roles in inducing apoptotic cell death through ROS-mediated pathways. ROS are generated at complexes I and III of ETC and are responsible, in conjunction with calcium levels, for the opening of the mitochondrial permeability transition pore, thereby leading to mitochondrial apoptotic pathways. This is followed by cytochrome c release into the cytosol with subsequent caspase-9 activation that ultimately activates caspase 3/7. To confirm the involvement of mitochondria we examined the activity of caspase 9 as an initiator of intrinsic apoptosis pathway. Thus we assayed the caspase 9 activity only after 6 h exposure. In this study, propofol induced the mitochondrial apoptotic pathway, as indicated by the increase in caspase activities as well as changes in mitochondrial function. Additionally, increased ROS are associated with decreased mitochondrial complex activity, and increased LDH release is associated with the loss of mitochondrial membrane potential during apoptosis [29]. The concentrations of propofol tested in this study varied from 12.5 to 150 μM. Plasma concentrations of propofol during anesthesia and sedation range between 2 (11 μM) and 5 μg/mL (27.5 μM) [30]. The concentration of propofol in tissues of rats treated with propofol at a dose of 20 mg/kg/h could reach 200 μM under certain conditions [31]. These reports justify the concentrations of propofol used in the present study. The duration of exposure used in this study ranged from 3 to 12 h, which was also within the clinically relevant period of exposure.

Although predictive factors for PRIS have not been established, there is a consensus that exposure to high doses of propofol for prolonged periods increases the risk factor of developing PRIS [1, 2, 8, 32]. In this study, we demonstrated that propofol at concentrations equal to or greater than 50 μM, but not at or below 25 μM, was cytotoxic within 6 h. However, 25 μM propofol significantly increased cell death and caspase activity after an incubation period of 12 h. In addition, propofol at 50 and 25 μM significantly suppressed OCR and increased ECAR within 6 and 12 h, respectively. At 100 μM, propofol inhibited both OCR and ECAR, which could be due to rapid cell death induced by propofol at this concentration. These results indicate that propofol at clinically relevant concentrations suppresses OXPHOS and induces a metabolic switch from OXPHOS to glycolysis, resulting in the enhancement of lactate production. This metabolic conversion can be one of the most critical cellular mechanisms of lactic acidosis observed in PRIS [1, 2].

Propofol-induced cell death has been reported [33–36]. In this study, we demonstrated that apart from neuronal SH-SY5Y cells, cells of other origins, such as C2C12 muscle cells, HeLa cervical carcinoma cells, and P29 lung cancer cells, are also susceptible to propofol-induced cell death. Although there is no consensus on the target organs or tissues involved in PRIS, our results suggest that propofol can exert toxicity against a wide range of tissues.

There are at least two known modes of cell death—apoptosis and necrosis. Apoptosis is a strictly regulated process involving the activation of specific proteases, which are responsible for the organized removal of damaged cells [37]. Necrosis is a physiologically different form of cell death, which is accompanied by the loss of mitochondrial membrane potential and an impairment of OXPHOS. In this study, propofol treatment promoted caspase-9 and caspase-3/7 activation. These data indicate that propofol activates the apoptosis pathway. Flow cytometry analysis indicated that the treatment of cells with propofol concentrations greater than 50 μM for 12 h also resulted in LDH release and increased the PI^+^/annexin V^−^ cell population, in addition to the PI^+^/annexin V^+^ cell population. These data strongly suggest that propofol elicits both types of cell death, resulting in cell membrane injury at 50 μM after 12 h of exposure. Propofol inhibited mitochondrial oxygen consumption in a time- and concentration-dependent manner. In this study, we measured OCR, which is dependent on the activity of each ETC complex in permeabilized cells, using an extracellular flux analyzer. Propofol is reported to inhibit the enzymatic activity of complexes II and IV in isolated mitochondria [38]. However, our study using cells indicated that propofol does not affect complex IV-dependent OCR. Propofol also induced ROS generation. Our findings strongly suggest that mitochondria play a critical role in this process. ROS production was not observed in ρ0P29 and P29mtΔ cells in response to propofol treatment. It has been demonstrated that impaired mitochondrial functioning leads to the increased production of ROS by ETC [21, 22, 39–41]. In fact, cybrids, such as P29mtA11 and P29mtB82M, with a mutation in the mitochondrial NADH dehydrogenase subunit 6 (*ND6*) gene generate ROS under 20% O_2_ conditions [14, 42], which implies that propofol-mediated cell death is dependent on ROS from mitochondria. Mitochondrial disease, once thought to be a rare clinical entity, is now recognized as an important cause of a wide range of neurological, cardiac, muscle, and endocrine disorders [4, 7]. It has been reported that complex I is uniquely sensitive to many anesthetic agents [43]. To evaluate this effect, we used cells harboring mtDNA mutations [13, 14, 44]. ρ0P29 cells, which do not have mtDNA, were resistant to 50 μM propofol. P29mtΔ cells, carrying the nuclear genome of P29 cells and the mitochondrial genome of ΔmtDNA4696, with a 4,696-bp deletion [13], were also resistant to propofol. However, P29mtA11 cells with the G13997A mutation in mtDNA and P29mtB82M cells with the 13885insC insertion, both of which affect the ND6 protein of complex I, are more sensitive to propofol than P29 cells. Propofol at 12.5 and 25 μM induced cell death and caspase-3/7 activation in both mutant cell lines within 6 h. Consistently, 25 μM propofol induced cell death in the presence of a sublethal concentration of rotenone. P29mtCOIM cells with a missense mutation in cytochrome c oxidase I (COI) are as sensitive to propofol as P29mtA11 and P29mtB82M cells. Accordingly, propofol induced cell death in the presence of oligomycin, indicating that cells with mutations in mtDNA are more sensitive to propofol.

In addition to the genetic mutations affecting mitochondrial function, biguanide-induced mitochondrial damage also induced caspase activation and cell death. Biguanides are primarily thought to inhibit respiratory complex I (NADH:ubiquinone oxidoreductase), which decreases ATP synthesis by OXPHOS [24, 25, 45, 46]. In addition, a number of studies have indicated that biguanides also affect complexes II–IV and F1F0-ATPase [26, 47]. Metformin and phenformin are used as antidiabetic drugs and are associated with lactic acidosis [26, 48, 49]. We found that both metformin and phenformin increased ECAR and decreased OCR. In addition to biguanides, other clinically used drugs, including chloramphenicol [50, 51], aspirin [52, 53], statins [24], and local anesthetics [17], also inhibit mitochondrial functions. Our results indicate that pre-exposure to mitochondrial inhibitors may increase the toxicity of propofol.

There are some limitations of this study. We tested propofol toxicity using cultured cells. We used mainly established cell lines from various tissue origins not but primary cultured cells. We focused on SH-SY5Y cells and P29 cells because SH-SY5Y cells were used in our precedent studies [16, 17] and transmitochondrial cybrids are available for P29 cells. Propofol induced cell toxicity in all the cell lines tested in the similar range of concentration. The evidence strongly suggested that inhibition of mitochondrial respiration is a general effect of propofol irrespective of cell type, and the findings might be applied to primary cultured cells with more physiological relevance in the study of PRIS. However, PRIS is a systemic syndrome. Thus studies on cells are not sufficient to completely understand the pathophysiology of PRIS. Studies featuring animal models are warranted to confirm our findings. It is reported that free propofol fraction in clinical conditions ranges from 1 to 3% at very low total propofol concentrations [54]. Although we performed our assays using 10% fetal calf serum the fraction of free propofol was not determined.

In conclusion, propofol used within a clinically relevant exposure time suppresses mitochondrial function, causes ROS generation, and induces a metabolic switch from OXPHOS to glycolysis, by targeting mitochondrial complexes I, II, and III *in vitro*. Our data also indicated that predisposition to mitochondrial dysfunction, caused by genetic mutations or the pharmacological suppression of ETC by biguanides, such as metformin and phenformin, promotes propofol-induced cell death and caspase activation. These mechanisms may constitute the molecular basis of PRIS.

## Supporting information

S1 Table Key resources table. Key resources used in this study was demonstrated.
(DOCX)

S2 Table Identification of pathogenic mutations in mtDNA sequences. Mutations in mtDNA sequences of hybrids were demonstrated.
(DOCX)

S3 Table Genetic characteristics of parent cells and their transmitochondrial cybrids. The characters of transmitochondrial cybrids were demonstrated.
(DOCX)

S1 Fig Propofol induced cell death in a concentration-dependent manner. SH-SY5Y cells were exposed to the indicated concentrations (12.5, 25, 50, 100, or 150 μM) of propofol for 6 h, after which they were harvested, and percentages of cell death were measured by flow cytometry. The ratio of PI-positive and/or annexin V-positive cells [(Q1 + Q2 + Q4)/(Q1 + Q2 + Q3 + Q4)] was used to calculate the percentage of dead cells.
(EPS)

S2 Fig Propofol decreased cell proliferation and increased caspase 3/7 activity. (A) SH-SY5Y cells were exposed to the indicated concentrations (12.5, 25, 50, 100, or 150 μM) of propofol for 6 h. The graphical depiction of levels of cell proliferation of treated and untreated cells, as evaluated by the MTS assay (n = 3) is shown. (B) SH-SY5Y cells were exposed to the indicated concentrations (12.5, 25, 50, 100, or 150 μM) of 2,4-diisopropylphenol for 6 h. The graphical depiction of caspase-3/7 activity (n = 3) is shown. Differences between treatment groups were evaluated by one-way ANOVA, followed by Dunnett’s multiple comparison test. *p < 0.05 compared to the control cell population (incubation for 0 h, no treatment).
(EPS)

S3 Fig Oxygen metabolism and ROS generation in SH-SY5Y cells treated with propofol. (A and C) Cell Mito Stress test profile indicating key parameters of mitochondrial oxygen consumption rate (OCR). (B and D) Cell glycolysis test profile indicating key parameters of the extracellular acidification rate (ECAR). OCR (A) and ECAR (B) in SH-SY5Y cells exposed to the indicated concentrations of propofol (50 or 100 μM) for 6 h were assayed by XFp extracellular flux analyzer™. (E–H) Sequential compound injections were performed to measure basal respiration, maximal respiration, non-mitochondrial respiration, and proton leak. OCR (basal respiration) (E), OCR (maximal respiration) (F), OCR (non-mitochondrial respiration) (G), and proton leak (H) in SH-SY5Y cells treated with 50 or 100 μM of propofol are shown. Data presented are expressed as the mean ± SD. Differences between results were evaluated by one-way ANOVA followed by Dunnett’s multiple comparison test *p < 0.05 compared to the control cell population.
(EPS)

S4 Fig Measurement of oxygen consumption in permeabilized cells. Activities of individual respiratory chain complexes were evaluated by employing specific substrates and inhibitors. (A) Cells were treated with a plasma membrane permeabilizer and supplemented with pyruvate and malate before measuring complex I-mediated respiration. Cells were sequentially treated with rotenone (complex I inhibitor), succinate (complex II substrate), antimycin A (complex III inhibitor), and TMPD plus ascorbate (complex IV substrate) as indicated. Oxygen consumption measurements were performed using an XFp extracellular flux analyzer. Distinct complex activities were calculated as follows: complex I-mediated respiration = (mean OCR value between points 1 and 2)—(mean OCR value between points 3 and 4); complex II-mediated respiration = (mean OCR value between points 5 and 6)—(mean OCR value between points 3 and 4); complex IV-mediated respiration = (mean OCR value between points 9 and 10)—(mean OCR value between points 7 and 8). (B) Representative traces of OCR indicating mitochondrial respiration using protocol A. (C) Cells were permeabilized as in protocol A, and treated with rotenone, followed by duroquinol as an electron donor at complex III. Complex III-mediated respiratory activity was calculated as (mean OCR value between points 7 and 9)—(mean OCR value between points 4 and 6). (D) Representative traces of OCR indicating mitochondrial respiration using protocol B.
(EPS)

S5 Fig Synergistic effect of propofol with the biguanide phenformin on caspase activity and cell death. Oxygen consumption rate (OCR) (A) and extracellular acidification rate (ECAR) (B) of SH-SY5Y cells exposed to indicated doses of phenformin (5 or 15 μM) for 6 h. SH-SY5Y cells were exposed to the indicated concentrations (25 or 50 μM) of propofol with or without treatment with 5 μM phenformin for 6 h. (C) Cells were harvested and cell death percentages were measured by flow cytometry. The ratio of propidium iodide (PI)-positive and/or annexin V-positive cells [(Q1 + Q2 + Q4)/(Q1 + Q2 + Q3 + Q4)] was used to calculate the percentage of dead cells (n = 3). (D) The graphical depiction of caspase-3/7 activity (n = 3) in each treatment group is shown. Data presented are expressed as the mean ± SD. Differences between results were evaluated by one-way ANOVA followed by Dunnett’s multiple comparisons test (A and B), or two-way ANOVA followed by Dunnfett’s multiple comparisons test (C and D). *p < 0.05 compared to the control cell population.
(EPS)

S1 Data Results of statistical analyses. Results of statistical analyses, including P-values were demonstrated.
(XLSX)
