# Supplementary material for: Propofol induces a metabolic switch to glycolysis and cell death in a mitochondrial electron transport chain-dependent manner
Source: PLoS One. 2018 Feb 15;13(2):e0192796. doi: 10.1371/journal.pone.0192796 (PMC5813975; doi:10.1371/journal.pone.0192796)
Supplement: S1 Table — Key resources used in this study was demonstrated. (DOCX) [file pone.0192796.s001.docx]

**S1 Table Key Resources Table**

| Reagents | Source | Identifier |
| --- | --- | --- |
| 2,6-diisopropylphenol (propofol) | Sigma–Aldrich | D126608 |
| 2,4-diisopropylphenol | Sigma–Aldrich | 557811 |
| oligomycin | Sigma–Aldrich | O4876 |
| carbonyl cyanide 4-(trifluoromethoxy) phenylhydrazone (FCCP) | Sigma–Aldrich | C2920 |
| rotenone | Sigma–Aldrich | R8875 |
| antimycin A | Sigma–Aldrich | A8674 |
| D-(-)-mannitol | Nacalai Tesque | 21303-32 |
| sucrose | Wako | 196-00015 |
| KH_2_PO_4_ | Wako | 169-04245 |
| MgCl_2_ | Nacalai Tesque | 20909-42 |
| HEPES | Nacalai Tesque | 17557-94 |
| EGTA | Nacalai Tesque | 37346-05 |
| albumin from bovine serum, fatty acid-free | Wako | 017-15141 |
| sodium pyruvate | Nacalai Tesque | 06977-34 |
| L-(-)-malic acid | Nacalai Tesque | 21030-44 |
| ADP, monopotassium salt | Wako | 303-50751 |
| Seahorse XF Plasma Membrane Permeabilizer | Agilent Technologies | 102504-100 |
| succinic acid, disodium salt | Nacalai Tesque | 32405-62 |
| tetramethylhydroquinone (duroquinol) | Tokyo Chemical Industry | T0822 |
| N,N,N′,N′-tetramethyl-*p*-phenylenediamine | Wako | 203-12821 |
| L-(+)-ascorbic acid, sodium salt | Nacalai Tesque | 03422-32 |
