# Supplementary material for: Propofol induces a metabolic switch to glycolysis and cell death in a mitochondrial electron transport chain-dependent manner
Source: PLoS One. 2018 Feb 15;13(2):e0192796. doi: 10.1371/journal.pone.0192796 (PMC5813975; doi:10.1371/journal.pone.0192796)
Supplement: S2 Table — Mutations in mtDNA sequences of hybrids were demonstrated. (DOCX) [file pone.0192796.s002.docx]

**S2 Table Identification of pathogenic mutations in mtDNA sequences**

| Position | Gene | Amino acid change | Mouse strain | Cell lines | | |
| --- | --- | --- | --- | --- | --- | --- |
|  |  |  | C57BL/6 | P29 | A11 | B82M |
| T6589C | *COI* | V421A | T | T | T | C |
| G9348A | *COIII* | V281I | G | G | G | A |
| 13885insC | *ND6* | Flame-shift | - | - | - | C |
| G13997A | *ND6* | P25L | G | G | A | G |

The G13997A mutation in *ND6* is a missense mutation that changes the amino acid proline to leucine at a site that is highly conserved throughout vertebrates. The 13885insC mutation in *ND6* is a frameshift mutation that has been previously reported as a pathogenic mutation inducing substantial complex I defects in some sublines of the L929 fibroblast cell line.

Bases: A, Adenine; G, Guanine; C, Cytosine; T: Thymine

Amino acids: V, Valine; A, Alanine; I, Isoleucine; P, Proline; L, Leucine
