# Supplementary material for: Propofol induces a metabolic switch to glycolysis and cell death in a mitochondrial electron transport chain-dependent manner
Source: PLoS One. 2018 Feb 15;13(2):e0192796. doi: 10.1371/journal.pone.0192796 (PMC5813975; doi:10.1371/journal.pone.0192796)
Supplement: S3 Table — The characters of transmitochondrial cybrids were demonstrated. (DOCX) [file pone.0192796.s003.docx]

**S3 Table Genetic characteristics of parent cells and their transmitochondrial cybrids**

|  |  | Fusion combinations | | |
| --- | --- | --- | --- | --- |
| Cell lines | Nuclear genotype | Nuclear donor | mtDNA donors |  |
| ρ0P29 | P29 | ρ0P29 | (−) |  |
| P29mtA11 | P29 | ρ0P29 | enA11 |  |
| P29mtB82M | P29 | ρ0P29 | enB82M |  |
| P29mtCOIM | P29 | ρ0P29 | enB82 |  |
| P29mtΔ | P29 | ρ0P29 | enB82mtΔ |  |

ρ0, mtDNA-less cells; en, enucleated donor.
